# Neoadjuvant modified TPF (docetaxel, cisplatin, fluorouracil) for patients unfit to standard TPF in locally advanced head and neck squamous cell carcinoma: a study of 48 patients

**DOI:** 10.18632/oncotarget.8934

**Published:** 2016-04-22

**Authors:** Jérôme Fayette, Clara Fontaine-Delaruelle, Alexis Ambrun, Clémentine Daveau, Marc Poupart, Antoine Ramade, Philippe Zrounba, Eve-Marie Neidhardt, Julien Péron, Alpha Diallo, Philippe Céruse

**Affiliations:** ^1^ Department of Medicine, Léon Bérard Center, University of Lyon, Lyon, France; ^2^ Department of Surgery, Croix-Rousse Hospital, University of Lyon, Lyon, France; ^3^ Department of Surgery, Edouard Herriot Hospital, University of Lyon, Lyon, France; ^4^ Department of Surgery, Léon Bérard Center, University of Lyon, Lyon, France; ^5^ Department of Medical Oncology, Institut du Cancer des Hospices Civils de Lyon, Centre Hospitalier Lyon-Sud, University of Lyon, Pierre-Bénite, France; ^6^ CNRS-UMR 5558, Biometry and Evolutive Biology Laboratory, Health and Biostatistics Team, Villeurbanne, France; ^7^ Department of Surgery, Lyon Sud Hospital Center, University of Lyon, Pierre-Bénite, France

**Keywords:** TPF, head and neck cancer, induction, cisplatin, frail patients

## Abstract

TPF (docetaxel, cisplatin, fluorouracil) is the standard chemotherapy used for induction in locally advanced head and neck squamous cell carcinoma (LAHNSCC). Its toxicity limits it to younger patients with good functional status and without significant comorbidity. Since modified TPF (mTPF) demonstrated higher tolerability with similar efficacy in gastric cancer, we tested this scheme on frail patients.

From July 2010 to July 2014, the files of the 48 patients treated for LAHNSCC with mTPF in three French institutions were retrospectively collected.

mTPF was chosen because of age>70 years, or severe denutrition, or PS>1, or severe comorbidities or after severe toxicity of standard TPF. During the first 4 cycles, 2 patients died, 14 secondary hospitalizations were required and 10 patients stopped treatment due to no lethal toxicity. Two patients died during radiotherapy.

The response rate was 83% (19% complete response). With a median follow-up of 15.2 months, 4 patients died during treatment, 8 died of non-head and neck cancer related disorders, 18 progressed (17 deaths) and 18 were free of disease. The median overall survival was 18.5 months (95% IC: 16.9-30.0).

mTPF is effective in terms of response rate compared with the standard TPF and could become a new option in induction for frail patients with LAHNSCC.

## INTRODUCTION

For locally advanced head and neck squamous cell carcinoma (LAHNSCC), radiotherapy potentiated by cisplatin remains a standard attested by a large meta-analysis [1]. Induction chemotherapy by PF (cisplatin and fluorouracil (5FU)) increases overall survival, but at a lower level than chemoradiotherapy. TPF (docetaxel, cisplatin and 5FU) as induction chemotherapy is superior to PF [2, 3]. Then, TPF followed by radiotherapy or chemoradiotherapy is a valid therapeutic option but still largely debated. All guidelines are ancient (ESMO guidelines were published in 2010) and do not take into account recent data. None of the four direct comparisons between TPF followed by radiotherapy and exclusive chemoradiotherapy could demonstrate any significantly superior strategy [4–7]. TPF is the gold standard for larynx preservation [8].

TPF is toxic and should be saved for patients in good general condition: performance status 0 or 1, no loss of weight > 10%, no severe comorbidities, age < 70 years.

Several of our patients met one of these criteria and could not receive TPF. Due to their fragility or to avoid mutilation, they were not operated on in first intent. Also due to their fragility, definitive chemoradiotherapy (even with cetuximab) was objected to by the radiotherapist. Radiotherapy alone is inferior to induction chemotherapy followed by radiotherapy. So we needed a specific induction chemotherapy.

In metastatic gastric cancer, TPF is a standard. Due to its toxicities, a randomized phase II study compared standard TPF (T and P 75 mg/m^2^ d1, F 750 mg/m^2^/d d1 to d5, every 3 weeks) and modified TPF (mTPF, T and P 40 mg/m^2^ d1, leucovorin 400 mg/m^2^ followed by a bolus of F 400 mg/m^2^ then 1000 mg/m^2^/d, d1-2, every 2 weeks). The tolerability was better with mTPF: with 6% *vs* 17% of febrile neutropenia, and 3% *vs* 20% of grade 3-4 nausea/vomiting. Furthermore, overall survival was in favor of mTPF with 18.8 months *vs* 12.6 months (*p* = 0.007) [9].

So after these promising results we decided to give mTPF to patients who had received standard TPF and experienced a great toxicity. We observed a good tolerability with apparently similar efficacy. We thus drafted a prospective phase II study and submitted it for public financing. However we were not selected and since all the drugs were generics, we could not obtain industrial financing. So we used this protocol for patients for whom a multidisciplinary team had chosen induction chemotherapy and who were unfit to standard TPF because we thought it better than palliative chemotherapy. Seeing these good results, we decided to collect the data of all our patients treated by mTPF in induction chemotherapy over a period of 4 years to show its tolerability and efficacy in fragile patients.

## PATIENTS AND METHODS

### Data collection

We retrospectively reviewed the files of all patients with histologically confirmed LAHNSCC for whom a decision of neoadjuvant chemotherapy was made by a multidisciplinary board but who were unfit for TPF. After the mTPF for gastric cancer was presented, they received mTPF in three institutions (Léon Bérard Center, Edouard Herriot Hospital, Croix-Rousse Hospital) between July 2010 and July 2014. Most patients had refused radical surgery at first, and were unfit for exclusive chemoradiotherapy. Tumors were classified using the UICC staging system [16].

Patient data were collected in accordance with the CNIL rules (the French authority for the protection of patient data) and kept anonymous.

### Treatment

mTPF consisted of docetaxel and cisplatin at 40 mg/m^2^ each on day 1, leucovorin 400 mg/m^2^ followed by a bolus of Fluorouracil (5FU) at 400 mg/m^2^ then 1000 mg/m^2^/d, d1-2, every 2 weeks. All patients received adequate antiemetic prophylaxis and prednisolone (50 mg, orally, twice a day for three days, starting on the morning before chemotherapy) to prevent hypersensitivity reactions and reduce docetaxel-related skin toxicity and fluid retention. Granulocyte colony-stimulating factor (G-CSF) was administered for primary prophylaxis, from day 4 for three days. No antibiotic was administered prophylactically. The number of planned cycles varied from 3 to 12.

After mTPF, and according to multidisciplinary decisions, patients underwent surgery (neck dissection and/or tumor surgery) followed by radiotherapy within three to seven weeks of completion of chemotherapy or surgery. Radiation was delivered over a seven-week period using conventional fractionation (total dose of 66 to 70 Gy). Radiotherapy was administered alone if the patient was judged unsuitable for potentiation or was potentiated with weekly cisplatin (40 mg/m^2^) or cetuximab (400 mg/m^2^ one week before radiotherapy, then 250 mg/m^2^ weekly).

### Assessment

Tumor responses were evaluated according to RECIST 1.1 criteria. Patients had cervical and thoracic CTs before treatment and after one or two months of chemotherapy. Patients with hypopharyngeal or laryngeal cancer underwent another panendoscopy.

### Statistical analysis

The response rate was estimated as being the proportion of patients who achieved complete or partial response out of the total number of patients who received at least one cycle of mTPF. Overall survival (OS) was defined as the time from the date of diagnosis to the date of death or to the date of the last follow-up visit for surviving patients (censored cases). Time to relapse (TTR) was defined as the time from the date of diagnosis to the date of recurrence. Survival estimates were calculated using the Kaplan-Meier method. The analysis was performed with SAS (version 9.2).

## RESULTS

### Patient characteristics

Between July 2010 and July 2014, 48 patients with histologically confirmed locally advanced or metastatic HNSCC were treated with mTPF in the three French institutions taking part in the study. Patient characteristics are summarized in Table 1. They were predominantly men (*n* = 41; 85%) with a median age of 59 years (range: 48-85) at onset of mTPF. For all these patients the multidisciplinary team recommended neoadjuvant chemotherapy. But they were unfit for standard TPF, due to age > 70 years (*n* = 10; 21%), PS > 1 (*n* = 19; 40%), loss of weight > 10% (*n* = 7; 15%) or severe comorbidities (*n* = 7; 15%: chronic obstructive pneumopathy, history of acute renal failure, concomitant rectal cancer, liver transplantation, adrenal insufficiency, severe arteriopathy, psychiatric disorders). Four patients had a sever toxicity after the first standard TPF (one febrile neutropenia, two colitis and one severe asthenia) and we decided to pursue with mTPF (*n* = 4; 8%). One patient received mTPF for unknown reason.

**Table 1:** Patient characteristics at onset of mTPF (*n* = 48)

	**N**	%
Median age, years [range]	59 [48-85]	
**Sex**		
Female	7	15
Male	41	85
**Tumor site at initial diagnosis**		
Oral cavity	8	17
Oropharynx	18	38
Hypopharynx	15	31
Larynx	6	12
Neck node without primary	1	2
**Tumor stage at initial diagnosis**		
II	1	2
III	6	13
IVa	29	60
IVb	12	25
**Intent of treatment**		
Operable patient, organ preservation	28	58
Inoperable patient, palliation	20	42
**PS at the onset of mTPF**		
0	12	25
1	17	35
2	16	33
3	3	6
**Reason for choice of mTPF instead of standard TPF**		
Age > 70 years	10	21
PS > 1	19	40
Loss of weight > 10%	7	15
Severe Comorbidities	7	15
Toxicity after a first cycle of standard TPF	4	8
Unknown	1	2

The two most common primary tumor sites were the oropharynx (*n* = 18; 38%) and the hypopharynx (*n* = 15; 31%). Twenty patients (42%) were judged inoperable. Overall, tumors were advanced at the time of diagnosis, with 29 (60%) and 12 (25%) patients with stage IVa or IVb tumors respectively.

### mTPF delivery and safety

Data on mTPF delivery and toxicities are summarized in Table 2. The vast majority of patients were scheduled to receive 4 cycles of chemotherapy and the median number of cycles administered was 4 (range 1-12). In total 214 cycles were administered.

**Table 2:** Delivery and toxicity of mTPF (median number of cycles: 4; total number of cycles: 214)

Toxicities	First cycle (*n* = 48)	Second cycle (*n* = 44)	Third cycle (*n* = 38)	Fourth cycle (*n* = 27)
Febrile neutropenia	2 (4%)	0	1 (3%)	1 (4%)
Diarrhea (gr 3-4)	3 (6%)	1 (2%)	1 (3%)	0
Secondary hospitalization	5 (10%)	4 (9%)	2 (5%)	3 (8%)
Transient creatinine elevation	4 (8%)	5 (11%)	2 (5%)	2 (5%)
Cause of discontinuation	4 interruptions for toxicity (8%)	1 death (2%) 4 interruptions for toxicity (8%)	1 death (3%) 2 interruptions for toxicity (5%)	Planned. No toxicity

Two patients (4%) died during chemotherapy and 10 patients (21%) stopped treatment due to toxicity. The levels of febrile neutropenia or grade 3-4 diarrhea were very low: for example 2 (4%) and 3 (6%) respectively for the first cycle. The numbers of secondary hospitalizations were 5 (10% of patients treated for this cycle), 4 (9%), 2 (5%) and 3 (8%) for the 4 first cycles respectively. For patients who pursued chemotherapy for more than 4 cycles, we did not observe particular toxicity.

### Efficacy of mTPF

The overall response rate according to RECIST intent-to-treat criteria was 83%, of which 19% were complete responses and 65% partial responses (Table 3); 10% of patients had stable disease, and 2% progressed on treatment.

**Table 3:** Best response to mTPF and type of treatment after induction

Best response to TPF			*N*	%
	**Overall response rate**		**40**	**83**
		Complete response	9	19
		Partial response	31	65
	Stable disease		5	10
	Progressive disease		1	2
	Not evaluable		2	4
		
**Surgery of the tumor**			**8**	**17**
	Mutilating		4	8
	Non-mutilating		4	8
			
**Node dissection** (of whom 14 had only node dissection)			**20**	**42**
		
**Radiotherapy**			**39**	**81**
	Exclusive		22	46
	Potentiated		17	35
		Weekly cisplatin	8	17
		Weekly cetuximab	6	12
		Unknown	3	6

### Treatments after mTPF

After mTPF, 8 (17%) patients had surgery, of whom 4 (7%) had non-mutilating surgery and 4 (8%) had mutilating surgery (due to insufficient response in operable patients). Neck dissection was performed on 20 (42%) patients because of persistent nodal disease (of whom 14 had only neck dissection). Indeed in France we prefer to perform node dissection if large involvement before radiotherapy. After mTPF, 39 (81%) patients were irradiated, 22 (46%) without potentiation, and 17 (35%) with potentiation. The type of potentiation was weekly cisplatin (40 mg/m^2^) for 8 patients and cetuximab for 6 patients (3 unknown). Four patients had temporary arrest of radiation (2 deaths, both potentiated −1 weekly cisplatin, 1 unknown- 2 others without potentiation) and all but the two dead received the planned cumulative dose of radiotherapy. In case of toxicity the potentation was stopped in order to favour the total dose of radiotherapy: 4/6 patients with cetuximab, 2/8 with weekly cisplatin and 1/3 with unknown potentation had to stop potentiation.

### Survival data

After a median follow-up of 15.2 months (range between 0.3 and 42 months), 18 (37.5%) patients relapsed and 17 died, and 18 patients were alive with no evidence of disease. Four died during treatment, and 8 died of non-head and neck cancer related disorders (2 cardiac failures, 2 secondary cancers - oesophagus, colorectal-, 3 unknown -probably cardiac failures, 1 anaphylaxis shock), due to the high fragility of these patients.

**Figure 1:**
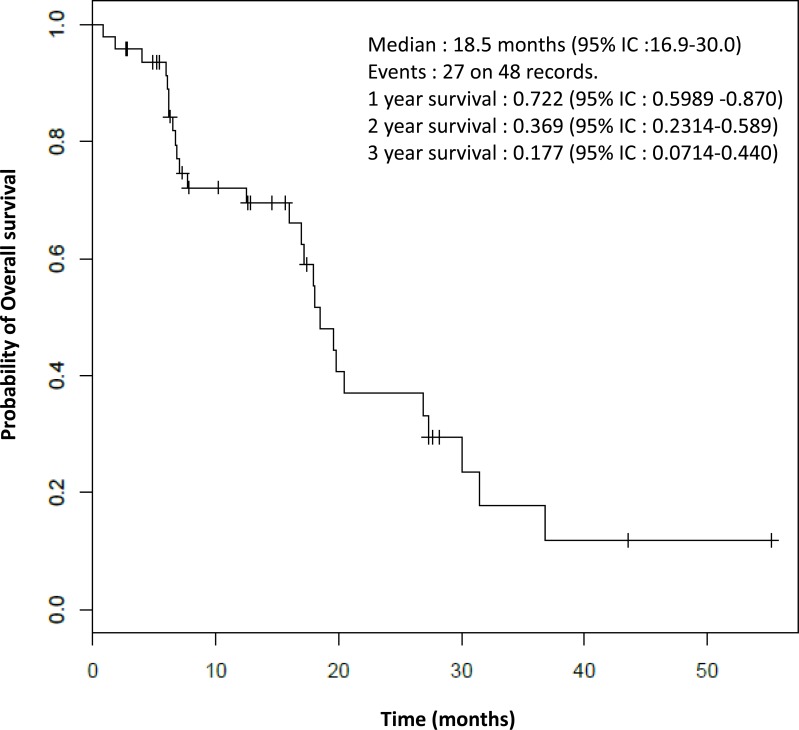
Overall survival

As shown in Figure 1, the median overall survival was 18.5 months (95% IC: 16.9-30.0).

**Figure 2:**
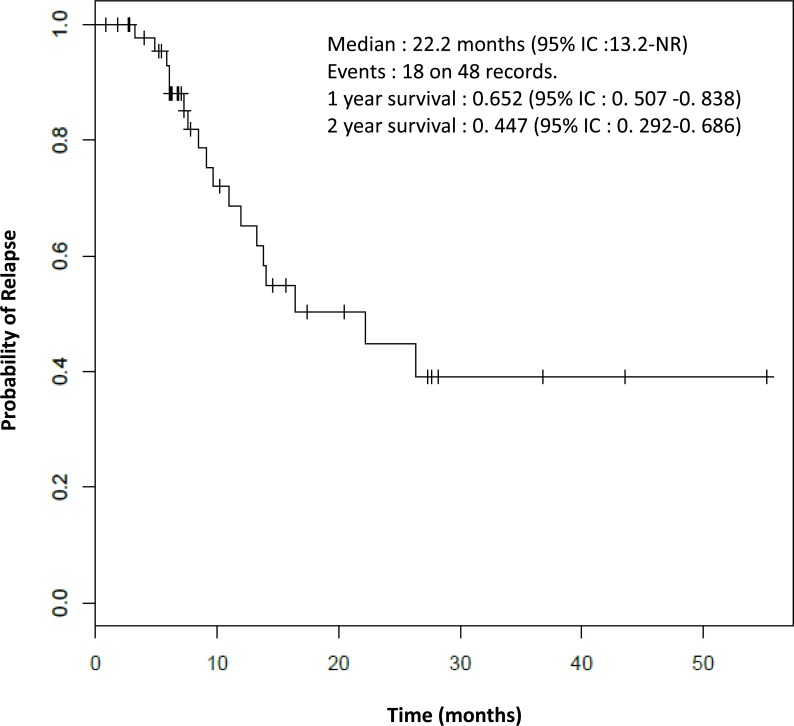
Time to relapse

Due to the frailty of these patients it seems more interesting to evaluate the median time to relapse instead of the progression-free survival. As shown in Figure 2, the median time to relapse was 22.2 months (95% IC: 13.2-NR).

## DISCUSSION

This retrospective study met its objectives and demonstrated tolerability and efficacy of mTPF in fragile patients with a LAHNSCC. Even if of course no direct comparison can be made between different studies, we should discuss our results in the light of the three pivotal phase III trials of standard TPF [2, 3, 10], and of our previously reported retrospective study of TPF by the same institutions in routine practice [11]. The patients were all PS 0-1 and had a median age of 53-57 years compared to 40% of patients who were at least PS 2 and had a median age of 59 years (and 21% of patients older than 70 years) in our study.

In terms of efficacy, at 83% the response rate of mTPF is similar to the 68-84% reported [2, 3, 10]. In terms of tolerability, with mTPF we observed only 2 toxic deaths (4%) and 4 febrile neutropenia (8%) despite 3 days of G-CSF. We did not use prophylactic ciprofloxacin because monotherapy with fluoroquinols induces a high level of bacterial mutations that lead to resistance and the expected duration of neutropenia is less than 7 days. On account of age and comorbidities, the use of G-CSF prophylaxis is recommended by ASCO guidelines but we probably chose too short a duration and for future treatments we will recommend at least 5 days of G-CSF. Because of our prudence with fragile patients treated by mTPF, we interrupted treatment for 10 (21%) of them (*vs* 6% with standard TPF). The rate of secondary hospitalization (29% *vs* 27%) was similar to that in our previous study with TPF for fit patients [11]. 3 out of 4 patients who experienced severe acute toxicity to TPF could receive mTPF safely. The continuation of treatment is not compromised by mTPF since 39 patients (82%) were irradiated, similar to other studies [2, 3, 10].

What is the optimal number of mTPFcycles? In the pivotal studies patients received 3 to 4 cycles of TPF with a cumulative dose of cisplatin and docetaxel of 225-300 mg/m^2^ for a duration of 2 to 3 months. So we propose 4 to 6 cycles of mTPF for the same length of time and doses ranging from 140 to 240 mg/m^2^. In our study, 5 and 3 patients received 8 and 12 cycles respectively on account of its good tolerability and efficacy in a context of severe comorbidities. So we decided to favour the quality of life and decided not to irradiate 5 patients and to delay the decision of radiotherapy for 3 others (radiation was finally performed after their health had generally improved).

The place of induction chemotherapy is still debated. Induction by TPF has demonstrated superiority to PF in terms of overall survival and laryngeal preservation and has become the standard treatment when induction is chosen [2, 3, 10, 12]. TPF is largely accepted for larynx preservation with an impressive level of larynx dysfunction-free survival at 10 years of 63,7% [8].

For other localizations or for inoperable tumors, chemoradiation with cisplatin remains the standard. But guidelines are ancient (ESMO guidelines were published in 2010) and do not take into account the most recent data. Clearly our population could not receive radiotherapy potentiated by cisplatin every three weeks at the dose of 100 mg/m2. Similarly our radiotherapists objected to potentiation by cetuximab (that was not directly compared to cisplatin) due to its toxicity in frail patients with gross tumors in our experience. Since induction chemotherapy is superior to radiation alone [13] we then preferred induction chemotherapy before radiation.

No study has yet demonstrated superiority of chemoradiation *versus* induction followed by radiotherapy (eventually with potentiation). Four phase III studies failed to draw a conclusion mainly because of methodological problems. In the first trial the per-protocol analysis showed superiority of induction followed by chemoradiation but did not reach a statistical significance as intent to treat, due to high levels of toxicity [7]. In two others, only 50% of the planned patients were included and finally no statistical difference could be shown [4, 6]. The last study compared four arms to take into account the role of cetuximab in combination with radiotherapy. Patients received TPF followed by chemoradiation or chemoradiation alone. For chemoradiation, patients received a potentiation by weekly cetuximab or by 2 cycles of cisplatin and 5FU. The data of patients irradiated with cisplatin or cetuximab were pooled to compare induction *versus* no induction: the median overall survival was significantly better with induction by TPF (53.3 months *vs* 30.3 months, HR = 0.72, 95% IC 0.55-0.96, *p* = 0.025) [5]. But since i) there is no direct demonstration of similar efficacy of potentiation by cisplatin or cetuximab, ii) the subgroup analysis of patients irradiated with cisplatin did not achieve significance, no definitive conclusion could be drawn.

In conclusion, our study demonstrated the safety of mTPF and its efficacy on response rate for patients unfit to TPF. mTPF could increase tolerability of induction chemotherapy for fit patients with similar efficacy and could increase compliance and efficacy of the entire sequence of treatment. Indeed induction proved superior to chemoradiation if the entire sequence could be administered [7]. These encouraging results lead us to a randomized study for fit patients comparing mTPF and TPF.
